# The occurrence and risk factors of bradycardia after the Maze procedure in patients with atrial fibrillation and tricuspid regurgitation

**DOI:** 10.1186/s13019-021-01653-1

**Published:** 2021-09-26

**Authors:** Xue Wang, Heng Gao, Chao Deng, Miaomiao Liu, Yang Yan

**Affiliations:** 1 Department of Cardiovascular Surgery, First Affiliated Hospital of Xi’an Jiaotong University, No. 277 Yanta West Road, Xi’an, 710061 People’s Republic of China; 2 Department of Emergency Internal Medicine, Shaanxi Provincial People’s Hospital, 256 Youyi West Road, Xi’an, 710068 People’s Republic of China

**Keywords:** Atrial fibrillation, Tricuspid regurgitation, The Maze procedure, Sinus bradycardia, Pulmonary hypertension

## Abstract

**Objective:**

To evaluate the occurrence and risk factors of bradycardia after the Maze procedure in patients with atrial fibrillation and tricuspid regurgitation.

**Methods:**

All patients underwent mitral valve (MV) replacement and concomitant bi-atrial cut-and-sew Maze procedure along with other cardiac surgical procedures were recruited from the Department of Cardiovascular Surgery at the First Affiliated Hospital of Medical College of Xi'an Jiaotong University. According to the severity of tricuspid regurgitation, all patients were divided into mild tricuspid regurgitation group and moderate-to-severe tricuspid regurgitation group. The general clinical data, biochemical indexes, intraoperative and postoperative data were collected. The relationship between tricuspid regurgitation and sinus bradycardia after the Maze procedure was analyzed by multivariate logistic regression model.

**Results:**

We enrolled 82 patients, including 24 males and 58 females. The patients had an average age of 56 ± 10 years old. There were 50 cases in mild tricuspid regurgitation group and 32 cases in moderate-to-severe tricuspid regurgitation group. Compared with the mild tricuspid regurgitation group, postoperative bradyarrhythmia (41% vs. 14%), pre-discharge bradyarrhythmia (63% vs. 14%), postoperative sinus bradycardia (34% vs. 10%) and pre-discharge sinus bradycardia (63% vs. 10%) in moderate-to-severe tricuspid regurgitation group were significantly increased (*P* < 0.01). In moderate-to-severe tricuspid regurgitation, the risk of sinus bradycardia increased after the Maze procedure (OR = 1.453, 95% CI 1.127–1.874), area under ROC curve was 0.81, the Jordan index was 0.665.

**Conclusion:**

The severity of tricuspid regurgitation may be an important factor affecting sinus bradycardia after the Maze procedure. It can be considered as a factor to predict sinus bradycardia after the Maze procedure.

## Introduction

Atrial fibrillation (AF) is one of the most common arrhythmias clinical practice. The incidence of AF increases with age. Atrial fibrillation is common in patients with valvular disease, which could further cause thromboembolism or even heart failure, seriously affecting the patient's quality of life, leading to high disability and mortality rates [1]. The Cox-Maze procedure (MP) is a well-established method of rhythm control in patients with AF, which has shown similar efficacy in patients with AF associated with valvular disease, coronary disease, or congenital heart disease compared with that in patients with AF alone [2–4]. In recent years, bipolar radiofrequency ablation, also known as Cox-Maze IV procedure (CM-IV), is widely used in the surgical treatment of AF [5–7]. However, the development of symptomatic bradyarrhythmia is a major complication of Cox-Maze IV procedure, which can lead to fatal or necessitate pacemaker implantation. Previous studies have reported a wide incidence of bradyarrhythmia with 2 to 21% during short-term follow-up after surgery [8–10]. Age, extended lesion set, and microwave energy source were identified as predictors of sinus node dysfunction or bradyarrhythmia. Sinus bradycardia (SB) has been shown to be the main type of bradyarrhythmia [8, 11–13].

Tricuspid regurgitation (TR) may be present in 10 to 50% of patients with mitral valve regurgitation or stenosis, or both, as well as other left-sided valve lesions [14–16]. It remains unknown that whether TR severity affects the incidence of SB after the Maze procedure. Therefore, we conducted this study to investigate the effect of the severity of TR on the incidence of SB in patients with atrial fibrillation after the Maze procedure.

## Methods

### Patients

All patients underwent the Cox-Maze IV procedure for persistent AF combined with TR between January 2015 and November 2020 were included. Patients with previous history of surgical cardiac surgery, paroxysmal atrial fibrillation along with sinus bradycardia, permanent pacemaker (PPM) implantation before the Cox-Maze IV procedure, malignant tumors, hematological diseases, severe liver and kidney diseases, children and pregnant women were excluded. All patients in this study had persistent AF, which was defined as continuous AF with more than 1 month duration before the Cox-Maze IV procedure and not self-terminating, in which cardioversion had not been indicated. Twelve-lead electrocardiographic (ECG) evidence of AF was checked at two or more separate visits at least 1 month after the operation date. This retrospective study was approved by our institutional review board, and the informed consent requirement could be avoided (No. XJTU1AF2020LSK-078).

### Surgical treatment

All patients underwent mitral valve (MV) replacement and concomitant bi-atrial cut-and-sew Maze procedure along with other cardiac surgical procedures at the discretion of cardiac surgery specialist. The decision on performing a tricuspid valve (TV) surgical intervention was made by cardiac surgery team, on the basis of the preoperative echocardiographic findings as well as on-site findings in the operation room.

### Study outcomes and definition

The outcome of this study was bradycardia development, the primary outcome was SB, defined as symptomatic sinus bradycardia associated with documented sinus exit block, pause, or arrest in any type of electrocardiographic data, including Twelve-lead ECG, Holter monitoring, or telemetry record. Transient asymptomatic postoperative junctional rhythm was not counted as a clinical event. The rate of high-degree atrioventricular block (AVB) and PPM implantation were defined as secondary outcomes.

The general clinical data, biochemical indexes, intraoperative and postoperative data were collected. According to the severity of TR, patients were divided into mild TR group and moderate-to-severe TR group. The occurrence of bradycardia including SB after the Cox-Maze IV procedure and the effect of TR and other effect factors on SB were evaluated.

### Definition of tricuspid regurgitation

The severity of TR was graded following previous study (Table 1) [2, 17]. The left ventricular ejection fraction (LVEF) was determined by simplified Simpson method, and the left ventricular end-diastolic diameter (LVEDD) was measured by M type ultrasound (Table 1).

**Table 1:** Recommendations for the echocardiographic assessment of tricuspid regurgitation: a summary from review of Arsalan M and the European Association of Cardiovascular Imaging

Parameters	Mild	Moderate	Severe
Tricuspid valve morphology	Normal	Normal/abnormal	Abnormal/flail/large coaptation defect
RA/RV/IVC dimension	Normal	Normal/enlargement	Enlargement
Area of backflow beam − central backflow beam (cm^2^)	< 5	5–10	> 10
VC width (cm)	Not defined	Not defined, < 0.7	> 0.7
PISA radius (mm)	≤ 0.5	0.6–0.9	> 0.9
Color flow TR jet	Small, central	Intermediate	Very large central jet or eccentric wall-impinging jet
CW signal of TR jet	Faint/parabolic	Dense/parabolic	Dense/triangular with early peaking (peak < 2 m/s in massive TR)

### Postoperative follow-up

Postoperative ECG was used to determine the presence of AF. ECG was continuously monitored during the patient’s stay in the intensive care unit, and the standard twelve-lead ECG was checked daily during the postoperative hospital stay. To evaluate cardiac function and the recovery of the atrial contraction, transthoracic echocardiography (TTE) was performed before discharge.

### Statistical analysis

All data were presented as frequencies and percent ages or mean ± standard deviant (SD). Differences between two groups were compared using unpaired Student’s t tests or Mann–Whitney U test for continuous variables and χ^2^ test for categorical variables. For laboratory results, we also assessed whether measurements were outside the normal range. All statistical analyses were performed using IBM Statistics 26. A two-tailed value of *P* < 0.05 was considered as statistically significant.

## Results

### Basic characteristics

Between January 2015 and November 2020, a total of 82 patients, including 24 men and 58 women were enrolled and had an average age of 56 ± 10 years old. All patients were divided into mild TR group and moderate-to-severe TR group, there were 50 cases in mild TR group and 32 cases in moderate-to-severe TR group.

Furthermore, according to whether SB occurs after the Maze procedure. The patients were divided into the patients with SB group and patients without SB group, there were 26 cases in the patients with SB group and 56 cases in patients without SB group. All patients underwent mitral valve (MV) replacement. The bi-atrial MP was performed in 82 patients. AV replacement was performed in 15 patients (18.3%), and left atrial volume reduction was performed in 28 patients (34.1%). Concomitant TV surgery was performed in 64 patients (78.0%), most of whom underwent TV repair (n = 60, 75.6%).

### SB after the Maze procedure

Within 1 h after operation, SB and AVB occurred in 16 (19.5%) and 1 (1.2%) patient, respectively. PPM was implanted in 3 patients (3.7%) with SB and in 1 patient (1.2%) with AVB. Compared with the mild TR group, moderate-to-severe TR group had a higher incidence of bradyarrhythmia (41% vs. 14%) and SB (34% vs. 10%) after the Maze procedure. Moreover, these patients also had a higher incidence of bradyarrhythmia (63% vs. 14%) and SB (63% vs. 10%) development before discharge compared with the mild TR group (*P* < 0.01, Table 2). The pulmonary systolic blood pressure of the patients with moderate-to-severe TR were significantly higher than those with the mild TR (41.75 ± 12.39 mmHg vs. 35.76 ± 9.04 mmHg) (*P* < 0.05, Table 3).

**Table 2:** Comparison of clinical characteristics according to the severity of TR in patients

Characteristics	Mild TR group (n = 50)	Moderate-to-severe TR group (n = 32)	*P* value
Age, y	58 (49–63)	58 (53–63)	0.875
Men, n (%)	16 (32)	8 (25)	0.497
Smoker, n (%)	9 (15)	6 (19)	0.932
Drinker, n (%)	3 (6)	0 (0)	0.875
Body mass index, kg/m^2^	24.00 ± 3.26	24.02 ± 3.59	0.985
Hypertension, n (%)	5 (10)	5 (16)	0.448
Diabetes, n (%)	2 (4)	2 (6)	0.645
ALT (U/L)	24.55 (17.5–44.2)	21.6 (13.5–32.25)	0.714
AST (U/L)	26 (20.25–30.75)	22 (18.05–30.725)	0.757
Total bilirubin (μmol/L)	15.6 (10.75–24.05)	18.2 (13.1–25.1)	0.063
Partial pressure of oxygen (mmHg)	85.65 (74.35–95.7)	85.8 (78.68–94.5)	0.827
Pro-BNP (ng/ml)	1452.5 (757.7–2594.26)	1314 (648.65–247.75)	0.112
LV ejection fraction (%)	60 (55–64)	58 (54–62)	0.682
Pulmonary systolic blood pressure (mmHg)	35.76 ± 9.04	41.75 ± 12.39	0.013
LV end-diastolic dimension (mm)	50.5 (47–53.25)	51.5 (47.75–56)	0.742
RV diameter (mm)	16 (15–18)	16 (15–18)	0.137
RV outflow tract diameter (mm)	26 (24–28)	27 (24–28)	0.935
AF duration time (years)	1.5 (0.2–6.5)	0.75 (0.14–2)	0.410
Preoperative heart rate	98 (79–114)	84 (71–102)	0.518
Euroscore II (%)	1.06 ± 0.36	1.13 ± 0.47	0.097

Data were expressed as mean ± SD unless otherwise indicated

*TR* tricuspid regurgitation, *ALT* alanine aminotransferase, *AST* aspartate aminotransferase, Pro-BNP, *LV* left ventricular, *RV* right valve, *AF* atrial fibrillation

**Table 3:** Comparison of intraoperative and postoperative characteristics according to the severity of TR in patients

Characteristics	Mild TR group (n = 50)	Moderate-to-severe TR group (n = 32)	*P* value
*Intraoperative index*			
Operation time (min)	255 (196.5–306.25)	237 (196.5–262.25)	0.309
Aortic cross-clamp time (min)	77 (56.75–93.5)	75.5 (56.75–88)	0.887
CPB (min)	112.44 ± 38.59	118.22 ± 32.59	0.485
*Postoperative index*			
Ventilator assistance time (d)	1 (1–2)	1 (0.95–1)	0.103
Detention time in ICU (d)	2 (2–4)	2 (2–3)	0.256
Length of stay (d)	20 (15–23)	21 (16.75–25.5)	0.181
Cerebral apoplexy (%)	0 (0)	1 (3)	0.209
Secondary tracheal intubation (%)	0 (0)	1 (3)	0.209
Infector (%)	3 (6)	1 (3)	0.555
Deliration (%)	1 (2)	2 (6)	0.317
*1 h after MP*			
Bradyarrhythmia (%)	7 (14)	13 (41)	0.006
SB (%)	5 (10)	11 (34)	0.007
AVB (%)	1 (2)	0 (0)	0.421
AF (%)	19 (38)	8 (25)	0.222
*Before discharge*			
Bradyarrhythmia (%)	7 (14)	20 (63)	0.000
SB (%)	5 (10)	20 (63)	0.000
AVB (%)	2 (4)	0 (0)	0.252
AF (%)	12 (24)	8 (25)	0.918
Pacemaker implanted (%)	1 (2)	3 (9)	0.130
Amiodarone (g)	5.6 (4.2–7.35)	5.5 (4.18–7.65)	0.977
Hospital deaths (%)	0 (0)	2 (6)	0.073
*Concomitant surgery*			
Concomitant TV surgery (%)	34 (68)	30 (94)	0.006
With or without TV	Without TV (n1 = 16) With TV (n1 = 34)	Without TV (n2 = 3) With TV (n2 = 29)	*P*1 = 0.544 *P*2 = 0.159
SB (%)	1 (6) 4 (12)	3 (100) 17 (59)	
Concomitant AV surgery (%)	11 (22)	4 (13)	0.278
Concomitant LA volume reduction (%)	16 (32)	12 (38)	0.608

Data were expressed as mean ± SD unless otherwise indicated

*CPB* cardiopulmonary bypass, *MP* maze procedure, *AVB* atrioventricular block, *LA* left atrial, *SB* sinus bradycardia, *P1* comparison of incidence of SB without TV surgery and with TV surgery in mild TR group, *P2* comparison of incidence of SB without TV surgery and with TV surgery in moderate-to-severe TR group

### Comparison of clinical date between patients with SB and without SB

The clinical data of patients with SB (n = 26) were compared with those of patients without SB (n = 56). Patients with SB were characterized by higher prevalence of hypertension (23% vs. 7%) and pulmonary systolic blood pressure (42.23 ± 11.08 mmHg vs. 36.57 ± 9.17 mmHg), larger tricuspid regurgitation area [6.2 (5.38–8.68) cm^2^ vs. 2.55 (1.5–4.5) cm^2^], longer hospital stay [22 (17.8–28) d vs. 19.5 (15–23) d], shorter operation time [240 (198–260) min vs. 255 (199–309) min] and ventilator assisted time (0.9 ± 0.5 d vs. 1.2 ± 0.7 d), lower the total amount of amiodarone before discharge [4.75 (2–6.28) g vs. 5.85(4.6–7.58) g] than those without SB (*P* < 0.05, Tables 4 and 5).

**Table 4:** Comparison of clinical characteristics according to whether SB development in patients

Characteristics	Without SB (n = 56)	With SB (n = 26)	*P* value
Age (years)	58 (52–63)	54 (51–64)	0.788
Men, n (%)	15 (27)	9 (35)	0.468
Smoker, n (%)	10 (18)	5 (19)	0.881
Drinker, n (%)	3 (5)	0 (0)	0.229
Body mass index, kg/m^2^	23.95 ± 3.34	23.65 ± 2.55	0.686
Hypertension, n (%)	4 (7)	6 (23)	0.040
Diabetes, n (%)	2 (4)	2 (8)	0.420
ALT (U/L)	24.5 (17–37.5)	17.5 (13–26.95)	0.122
AST (U/L)	24.5 (18.25–30.75)	21 (17.75–27.75)	0.247
Total bilirubin (μmol/L)	16.85 (12.13–23.38)	13.8 (11.35–23.68)	0.458
Partial pressure of oxygen (mmHg)	85.35 (74.45–94.43)	88.2 (79.55–94.5)	0.397
Pro-BNP (ng/ml)	1452.5 (753.9–2435.75)	1181 (572.3–2086.25)	0.140
LV ejection fraction (%)	58.52 ± 8.40	59.71 ± 5.43	0.510
Pulmonary systolic blood pressure (mmHg)	36.57 ± 9.17	42.23 ± 11.08	0.017
LV end-diastolic dimension (mm)	51 (46.25–54)	51 (46–55.25)	0.747
RV diameter (mm)	25.66 ± 67.06	17 ± 4.28	0.514
RV outflow tract diameter (mm)	25.96 ± 4.65	25.77 ± 2.85	0.844
TR area (cm^2^)	2.55 (1.5–4.5)	6.2 (5.38–8.68)	0.000
AF duration time (y)	2 (0.2–5)	0.5 (0.1–2)	0.176
Preoperative heart rate	84 (70–102)	94 (75–111)	0.250
Euroscore II (%)	1.09 ± 0.38	1.09 ± 0.46	0.159

**Table 5:** Comparison of intraoperative and postoperative characteristics according to whether SB development in patients

Characteristic	Without SB (n = 56)	With SB (n = 26)	*P* value
*Intraoperative index*			
Operation time (min)	255 (199–309)	240 (198–260)	0.070
Aortic cross-clamp time (min)	80 ± 26	77 ± 27	0.625
CPB (min)	112 (92–138)	110 (92–148)	0.865
*Postoperative index*			
Ventilator assistance time (d)	1.2 ± 0.7	0.9 ± 0.5	0.037
Detention time in ICU (d)	2 (2–4)	2 (2–2)	0.121
Length of stay (d)	19.5 (15–23)	22 (17.8–28)	0.047
Cerebral apoplexy	0 (0)	1 (4)	0.420
Secondary tracheal intubation	1 (2)	0 (0)	0.493
Infector (%)	3 (5)	1 (4)	0.768
Amiodarone (g)	5.85 (4.6–7.58)	4.75 (2–6.28)	0.016
Hospital deaths (%)	0 (0)	2 (8)	0.768
*Concomitant surgery*			
Concomitant TV surgery (%)	42 (72)	22 (85)	0.328
Concomitant AV surgery (%)	11 (20)	4 (15)	0.643
Concomitant LA volume reduction (%)	22 (39)	8 (31)	0.456

### The association of TR and SB after the Maze procedure

**Fig. 1:**
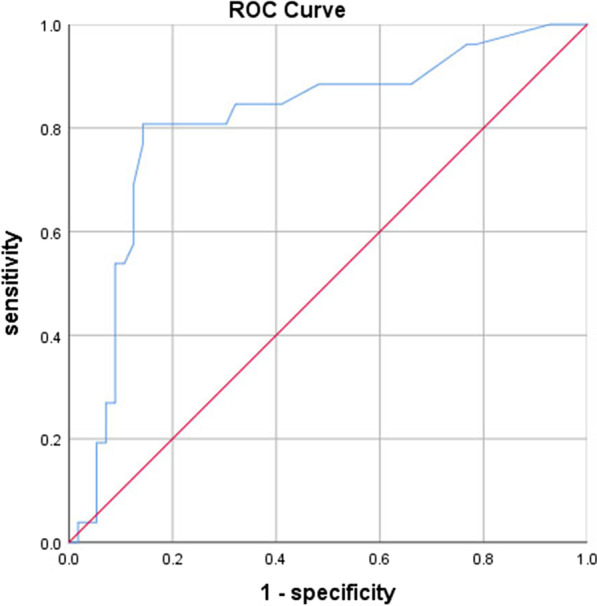
Roc curve of tricuspid regurgitation area in predicting sinus bradycardia after operation

We analyzed the relationship between tricuspid regurgitation and SB after the Maze procedure by multivariate logistic regression model. In moderate-to-severe TR group, risk of SB increased after the Maze procedure (OR = 1.453, 95% CI 1.127–1.874, Table 6). Area under ROC curve was 0.81, the predictive sensitivity was 80.8%, the specificity was 85.7% (Fig. 1), the accuracy was 84.1%, the Jordan index was 0.665, the positive predictive value was 78.8% and the negative predictive value was 87.8%.

**Table 6:** Predictors of recurrent SB after MP

Characteristics	B	S.E	Wald value	*P* value	OR	95% CI
TR area	0.374	0.130	8.317	0.004	1.453	1.127–1.874
Operation time	− 1.126	1.040	1.172	0.279	0.324	0.042–2.491
Ventilator assistance time	− 0.107	1.026	0.011	0.917	0.899	0.120–6.709
length of stay	− 1.064	0.797	1.782	0.182	0.345	0.072–1.645
Amiodarone	0.000	0.000	0.428	0.513	1.000	0.999–1.000
Pulmonary systolic blood pressure (mmHg)	0.087	0.054	2.626	0.105	1.091	0.982–1.213
Concomitant TV surgery	0.030	0.046	0.427	0.513	1.030	0.942–1.127
Concomitant AV surgery	0.074	0.061	1.434	0.231	1.076	0.954–1.214
Concomitant LA volume reduction	− 0.098	0.116	0.711	0.399	0.907	0.723–1.138
Pro-BNP	− 0.007	0.081	0.008	0.927	0.993	0.847–1.163
Preoperative heart rate	0.021	0.015	2.012	0.156	1.021	0.992–1.050
AF duration time	− 0.006	0.006	0.880	0.348	0.994	0.982–1.006
LVEF	− 1.526	0.965	2.499	0.114	0.217	0.033–1.442
LVEDD	0.064	0.032	4.083	0.043	1.066	1.002–1.134
RV diameter (mm)	− 0.291	0.159	3.350	0.067	0.748	0.547–1.021
RV outflow tract diameter (mm)	0.374	0.130	8.317	0.004	1.453	1.127–1.874

## Discussion

Cox-Maze IV is considered as an effective treatment on the control of AF. SB is a major complication of Cox-Maze IV. Most previous studies have focused on the efficacy of the MP. However, data about safety outcomes, especially the natural course from a chronological viewpoint, are lacking, and there are few studies on the occurrence rate and risk factors of SB after the Maze procedure [18, 19]. Whether TR was associated with the occurring of SB after MP is still unknown. Cho et al. [8] has evaluate the incidence of sick sinus syndrome (SSS) after the Maze procedure with mitral valve surgery, and showed only that the proportion of moderate-to-severe TR were different between patients with SSS and patients without SSS.

This retrospective analysis focused on the evaluation on the occurrence of bradycardia after the Maze procedure in patients with AF and TR. The effect of TR and other factors on bradycardia was also investigated. Firstly, according to severity of TR, We found that patients with moderate-to-severe TR had a higher incidence of bradyarrhythmia than those with the mild TR after the Maze procedure, and had a higher incidence of bradyarrhythmia and SB than those with the mild TR before discharge. The pulmonary systolic blood pressure of the patients with moderate-to-severe TR was significantly higher than those with the mild TR. Thus, we suspect that the possible mechanism is that AF causes the atrial muscle to lose normal electrophysiological conduction, atrial activity disorder, blood inability to fill and expel, blood stasis in the atrium, leading to atrial enlargement, tricuspid annulus retraction and dilatation, and right ventricular enlargement. With further expansion of the right ventricle, tricuspid leaflets are incompatibility and regurgitation also aggravate [20–24]. With the progress of the disease, pulmonary hypertension was induced, the level of 5-HT increased in the pulmonary circulation, which caused pulmonary vasoconstriction, exacerbation of fibrosis and remodeling of the right heart system, resulting in sinus node dysfunction, autonomic nervous tension imbalance, jugular vein irritation, gastrointestinal dysfunction and other complications, while jugular vein irritation, gastrointestinal dysfunction could cause autonomic nervous dysfunction, and these further decrease sinus node function [25, 26].

Most previous studies thought that age, extended lesion, and microwave energy source were identified as predictors of sinus node dysfunction or SB, however, these observations are insufficient to change the clinical practice [8, 11–13]. Our study has used a fixed microwave energy source, excluded the confounding factors including age and cardiac enlargement, to investigate other factors affecting the incidence of SB after the Maze procedure. According to whether SB occurred after the Maze procedure, the patients were divided into the patients with SB and patients without SB. Interestingly, comparing with the patients without SB, pulmonary systolic blood pressure and tricuspid regurgitation area significantly increased in the patients with SB. Furthermore, the multivariate logistic regression showed that the severity of TR is an important factor affecting the incidence of SB after the Maze procedure.

Because it was a retrospective study, all patients underwent preoperative ultrasound evaluation. Patients with mild tricuspid regurgitation underwent left ventricular valve replacement during operation. Tricuspid valvuloplasty was performed in patients with high-risk factors such as atrial fibrillation or tricuspid valve ring diameter greater than 40 mm.

There were some limitations in this study. The sample size is too small. The study groups are not matched or adjusted. There are unaccountable selection biases without precising detailed rhythm status and preoperative rhythm medication of the patients. Whether TV repair in the setting of MAZE procedure could decrease the occurrence of SB, AVB or PPI is worth to be further investigated.

## Conclusion

In conclusion, severity of TR was considered as an indicator for the incidence of SB after the Maze procedure.

## Data Availability

Not applicable.

## Abbreviations

AF: Atrial fibrillation
MP: Maze procedure
CM-IV: Cox-Maze IV
SB: Sinus bradycardia
TR: Tricuspid regurgitation
PPM: Permanent pacemaker
ECG: Electrocardiographic
MV: Mitral valve
TV: Tricuspid valve
AVB: Atrioventricular block
LVEF: Left ventricular ejection fraction
LVEDD: Left ventricular end-diastolic diameter
TTE: Transthoracic echocardiography
SD: Standard deviant
MV: Mitral valve
SSS: Sick sinus syndrome
